# Highly Sensitive Measurement of Oxygen Concentration Based on Reflector-Enhanced Photoacoustic Spectroscopy

**DOI:** 10.3390/s22145087

**Published:** 2022-07-06

**Authors:** Shuhan Yang, Shunda Qiao, Xiaonan Liu, Yufei Ma

**Affiliations:** National Key Laboratory of Science and Technology on Tunable Laser, Harbin Institute of Technology, Harbin 150001, China; yangshuhan@stu.hit.edu.cn (S.Y.); 20b921022@stu.hit.edu.cn (S.Q.); 21b921010@stu.hit.edu.cn (X.L.)

**Keywords:** photoacoustic spectroscopy (PAS), wavelength modulation spectroscopy (WMS), oxygen (O_2_) gas detection, reflector-enhanced

## Abstract

Oxygen (O_2_) is a colorless and odorless substance, and is the most important gas in human life and industrial production. In this invited paper, a highly sensitive O_2_ sensor based on reflector-enhanced photoacoustic spectroscopy (PAS) is reported for the first time. A diode laser emitting at 760 nm was used as the excitation source. The diode laser beam was reflected by the adopted reflector to pass thorough the photoacoustic cell twice and further increase the optical absorption. With such enhanced absorption strategy, compared with the PAS system without the reflector, the reflector-enhanced O_2_-PAS sensor system had 1.85 times the signal improvement. The minimum detection limit (MDL) of such a reflector-enhanced O_2_-PAS sensor was experimentally determined to be 0.54%. The concentration response of this sensor was investigated when O_2_ with a different concentration was used. The obtained results showed it has an excellent linear concentration response. The system stability was analyzed by using Allan variance, which indicated that the MDL for such a reflector-enhanced O_2_-PAS sensor could be improved to 318 ppm when the integration time of this sensor system is 1560 s. Finally, the O_2_ concentration on the outside was continuously monitored for 24 h, indicated that this reflector-enhanced O_2_-PAS sensor system has an excellent measurement ability for actual applications in environmental monitoring, medical diagnostics, and other fields.

## 1. Introduction

Oxygen (O_2_) is a colorless and odorless substance, and is the most common gas in human life and industrial production. Because of its combustibility, O_2_ in high concentration too easily causes explosions after mixing with other dangerous gases. In industrial production, O_2_ concentration monitoring is not only conducive to the complete combustion of fuel, and reduces exhaust emissions, but is also related to smooth production activities and workers’ safety. In food processing, the existence of O_2_ makes aerobic microorganisms multiply and grow, causing an oxidation reaction, which will reduce the shelf life and quality of food. In the medical field, the detection of blood O_2_ concentration in the human body can be used to monitor the physiological status of patients over time. Therefore, the detection of O_2_ concentration has a very high significance in medical [1], industry, life [2], and other fields.

With these broad requirements in applications, O_2_ sensors have been developed vigorously in recent years. Electrochemical devices [3,4], optical O_2_ sensors [5,6], and luminescent dissolved O_2_ sensors [7] based on fluorescence quenching [8] are typically used in O_2_ detection. The electrochemical O_2_ sensor device is widely adopted because of its low price and small size. However, long-term contact with the substance under test can cause corrosion of the device. Optical sensors have the merits of high sensitivity, long life and noninvasive detection [9,10,11,12]. Tunable diode laser absorption spectroscopy (TDLAS) is one of the widely used optical methods for gas detection [13]. However, due to its non-zero background measurement, the detection performance is restricted. Furthermore, the photodetector has wavelength response limitations, so it is not suitable for measurement in all wavebands.

**Figure 1:**
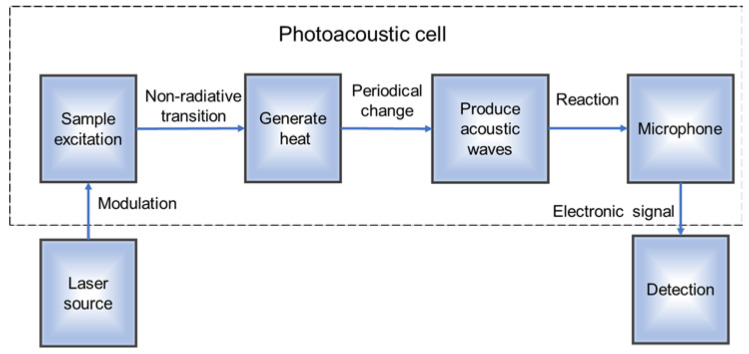
Schematic diagram of the principle of PAS.

With the appearance of highly sensitive microphones [14] and piezoelectric ceramic microphones, photoacoustic spectroscopy (PAS) has developed rapidly as a popular method to detect gas concentration in recent years [15,16,17,18]. It has the advantages of high sensitivity, high selectivity, zero background, and real-time online monitoring [19]. As shown in Figure 1, the principle of PAS is that when a substance absorbs light and receives excitation, it can return to its initial state by non-radiative transition. Because the absorbed light intensity changes periodically, a pressure wave is generated in the sample. Since the frequency of the modulated light is generally in the acoustic range, these pressure fluctuations transform into acoustic waves and can be detected by acoustic wave detectors. The intensity of the detected photoacoustic signal is proportional to the sensitivity of the microphone, the power of the light source, the gas concentration, and the absorption coefficient [20,21].

In this invited paper, a reflector-enhanced PAS-based O_2_ sensor was presented for the first time. A plane mirror coated with gold film was used as the reflector to reflect the diode laser beam and increase the absorption time. Using this strategy, the optical absorption and sensor signal level were improved. A wavelength modulation spectroscopy (WMS) technique combined with second harmonic signal demodulation (2*f*) was used to simplify the data processing and further improve the sensor performance.

## 2. Experimental Setup

**Figure 2:**
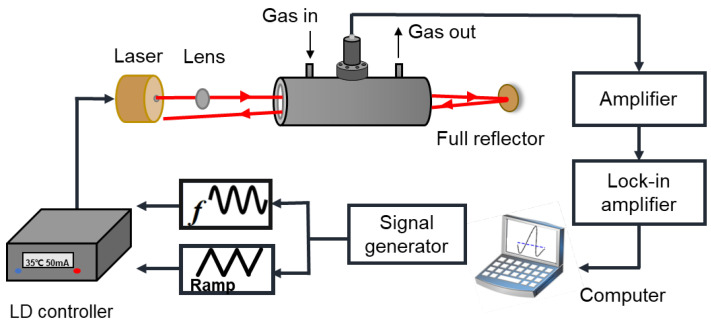
Diagram of the reflector-enhanced O_2_-PAS experimental system.

The experimental setup of the reflector-enhanced O_2_-PAS system is shown in Figure 2. The laser source is a diode laser with an output wavelength of 760 nm (Eblana Photonics, model: EP760-DM-TP39, Dublin, Ireland). Firstly, the laser beam passes through a lens for collimation. After that, it passes through a resonant photoacoustic cell to generate the photoacoustic signal. The resonant PA cell has a central cylindrical tube as an acoustic resonator and its performance depends on Q factor and cell constant. The PA cell operates in the first longitudinal resonance mode, where the Q factor increases with the radius of the PA cell. However, the cell constant decreases with the increase in the PA cell radius. For length consideration, the element constant increases with the increase in PA element length, but the resonance frequency decreases with the increase in the PA element length. The diameter and length of the cylindrical acoustic resonators are 10 mm and 100 mm, respectively. Two buffers with radius of 25 mm and 50 mm are fixed on both sides of the resonator to reduce noise generated by airflow and interference signals generated by window absorption. Two calcium fluoride (CaF_2_) windows are attached at both ends of the PA cells. The acoustic signal in the PA cell is ultimately detected by a condenser microphone with a detection sensitivity of 50 mV/Pa. A plane mirror coated with gold film is placed at the exit of the photoacoustic cell to serve as the reflector. The reflected laser passes through the photoacoustic cell again to increase the laser absorption. In the experiment, O_2_ and pure nitrogen (N_2_) were mixed to obtain 20–80% O_2_ concentration, and the flow rate was controlled through the gas mass controller. The generated photoacoustic signal was detected by a microphone. This signal was amplified by a preamplifier, and finally demodulated by a lock-in amplifier to obtain the second harmonic (2*f*) signal.

**Figure 3:**
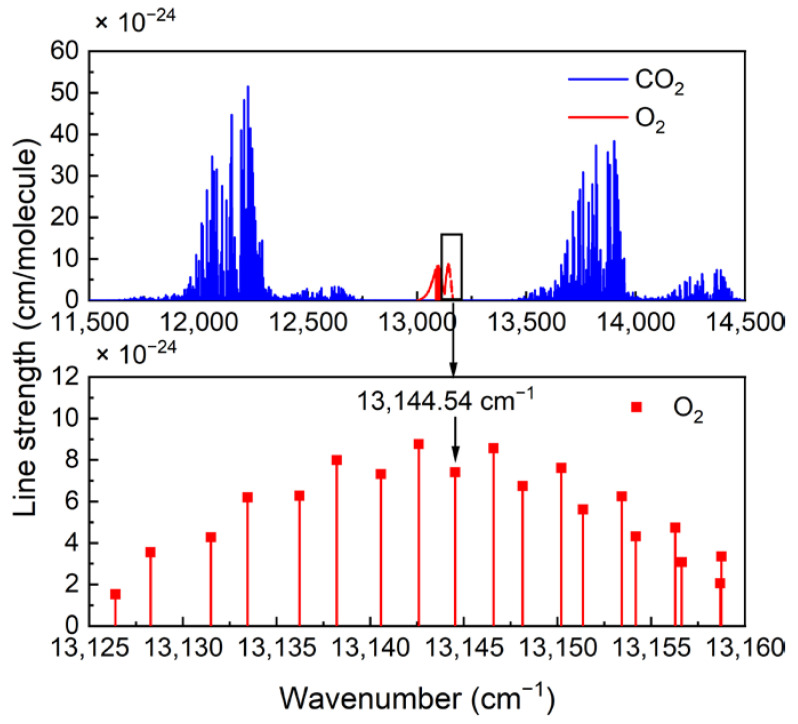
Absorption lines calculated based on the HITRAN2020 database.

In order to improve the intensity of the photoacoustic signal, it is necessary to optimize the O_2_ absorption line. Absorption spectrum lines calculated based on the HITRAN2020 database are shown in Figure 3. In the range of 13,000–13,200 cm^−1^, only O_2_ absorption lines exist, which means there is no interference from other gases. In addition, lasers in this band are available on the market. Therefore, in the experiment, a strong absorption line located at 13,144.54 cm^−1^ was selected.

## 3. Experimental Results and Discussions

**Figure 4:**
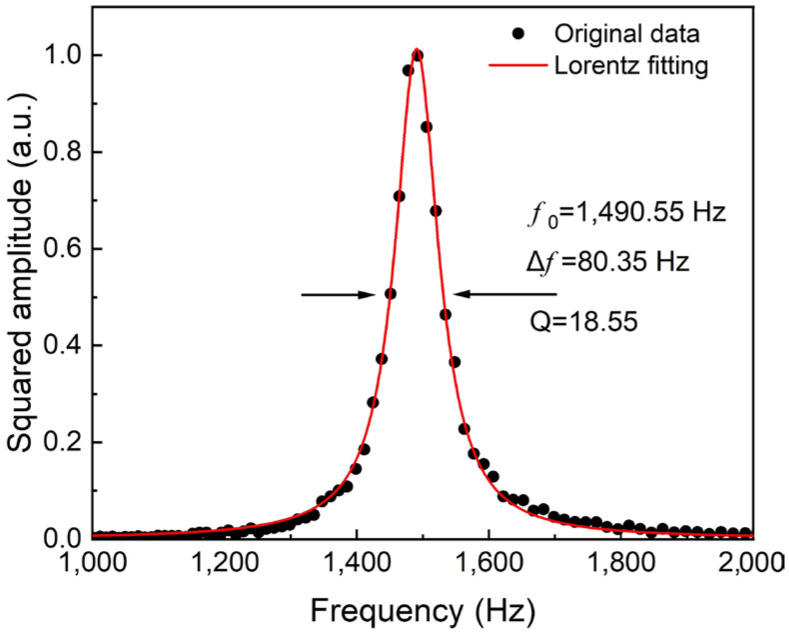
The resonant characteristic of the photoacoustic cell.

**Figure 5:**
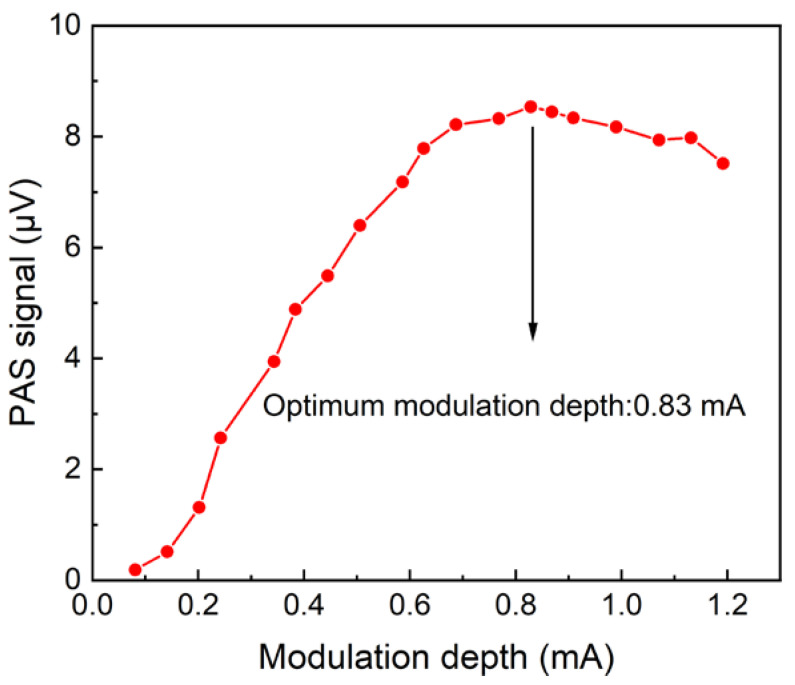
The relationship between O_2_-PAS signal amplitude and modulated current under the condition of O_2_ concentration of 20%.

Firstly, the resonant frequency characteristic of the photoacoustic cell used was measured. The obtained curve was fitted by Lorentz function and the results are shown in Figure 4. The resonance frequency *f*_0_ was found to be 1490.55 Hz, and the width Δ*f* at the half-high value was 80.35 Hz. According to *Q* = *f*_0_/Δ*f*, the quality factor *Q* was calculated to be 18.55. In this experiment, the demodulation frequency of the lock-in amplifier was set as *f*_0_/2 = 745.27 Hz to ensure the maximum 2*f* signal amplitude. In WMS, the amplitude of the sensor signal is affected by the laser modulation depth. Therefore, in the following experiments, the relationship between the modulation depth and PAS signal level was investigated. When the O_2_ concentration was 20% and the modulation depth was 0-1.2 mA, the amplitude of the 2*f* signal was measured, and this is shown in Figure 5. It can be seen that when the modulation depth was 0.83 mA, the PAS signal reached the maximum. When the modulation depth was greater than 0.83 mA, the signal value gradually decreased. Therefore, 0.83 mA was chosen as the optimum modulation depth in subsequent experiments.

**Figure 6:**
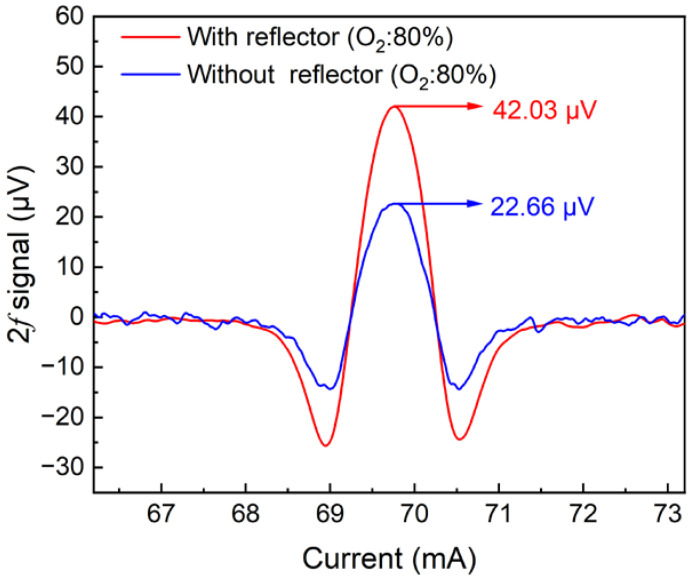
O_2_-PAS 2*f* signal after the addition of reflector under the condition of O_2_ concentration of 80%.

In the experiment, the signal generator produced a sawtooth wave with an 8 mV amplitude. The bias current of the laser was 67.2 mA, and thus the scanning current could be controlled to 67.2–73.6 mA. The current and temperature of the laser controller at the absorption line were 70 mA and 35 °C, and the laser output power was 14.8 mW under this condition. In this condition, the output wavelength of the laser swept through the absorption line of 13,144.54 cm^−1^ completely. A sinusoidal wave with frequency *f* = *f*_0_/2 = 745.27 Hz was superimposed on the sawtooth current to perform the wavelength modulation. The PAS signal was detected by the microphone and transmitted to the lock-in amplifier. The lock-in amplifier can filter out the jamming interference of other frequency signals. Its integration time was set to 1 s. To increase the optical absorption, a gold-coated reflector was added in the experiment to make the laser pass through the photoacoustic cell twice. Figure 6 shows the 2*f* signal measured with the addition of the reflector when the O_2_ concentration was 80%. It can be seen that the signal amplitude was increased by 1.85 times after the reflector was added.

**Figure 7:**
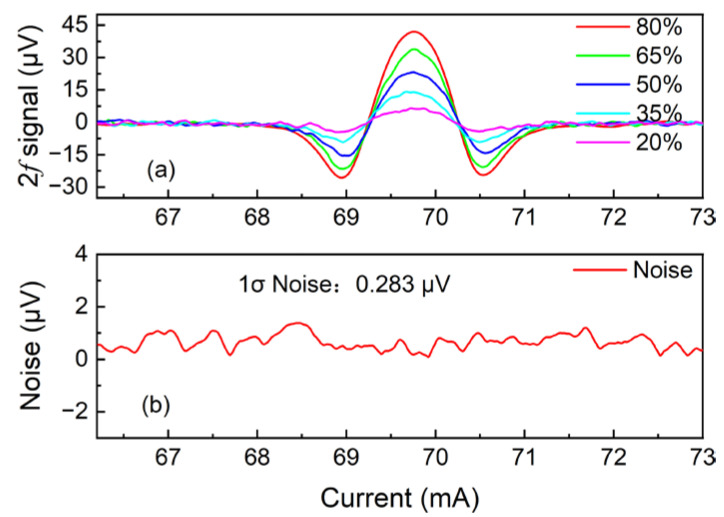
(**a**) 2*f* signal of reflector-enhanced O_2_-PAS sensor at different O_2_ concentrations; (**b**) the noise of the system when pure N_2_ is adopted.

**Figure 8:**
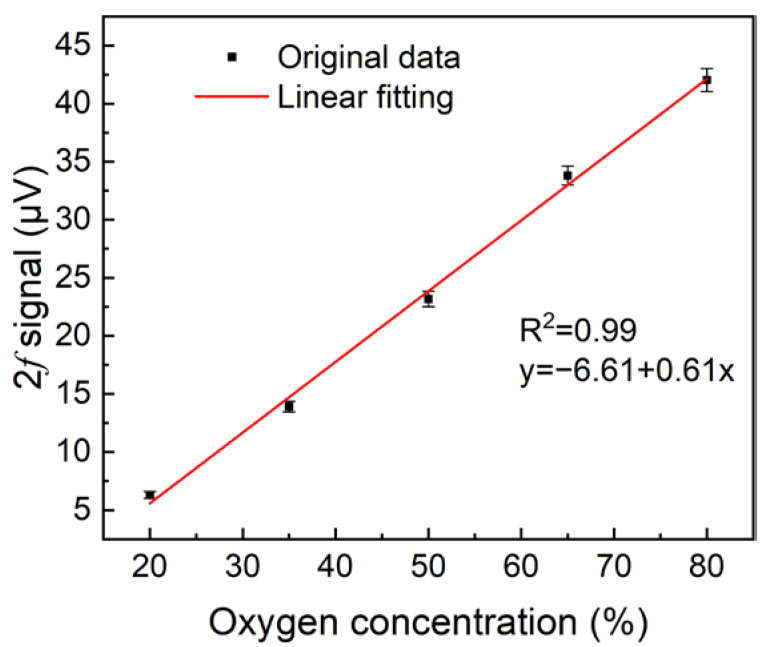
Linear fitting of 2*f* signal peak values at different O_2_ concentrations.

In order to verify the linear response of the reflector-enhanced O_2_-PAS system to O_2_ concentration, the 2*f* signal at different O_2_ concentrations was measured and averaged 20 times, and the final results are shown in Figure 7a. In this investigation, two mass flow controllers were used to control the gas flow rate of 80% O_2_ and pure N_2_, respectively, to produce an O_2_ mixture with different concentrations of 20%, 35%, 50%, 65%, and 80%. The linear fitting of the obtained O_2_-PAS 2*f* signal amplitude with O_2_ concentration is shown in Figure 8. The calculated R squared value is R^2^ = 0.99, indicating that this O_2_-PAS sensor has a good linear response to the O_2_ concentration level.

The noise level of this O_2_-PAS sensor was determined when the photoacoustic cell was filled with pure N_2_. The measured result is shown in Figure 7(b). The standard deviation of this measurement is 1σ = 0.283 μV. When the selected oxygen concentration is 65% the measured 2*f* signal is 33.803 μV and the SNR is 119. According to the formula minimum detection limit (MDL) = O_2_ concentration/SNR, the MDL of this reflector-enhanced O_2_-PAS sensor system is finally calculated to be 0.54%. Without the reflector, the MDL is 0.99%.

**Figure 9:**
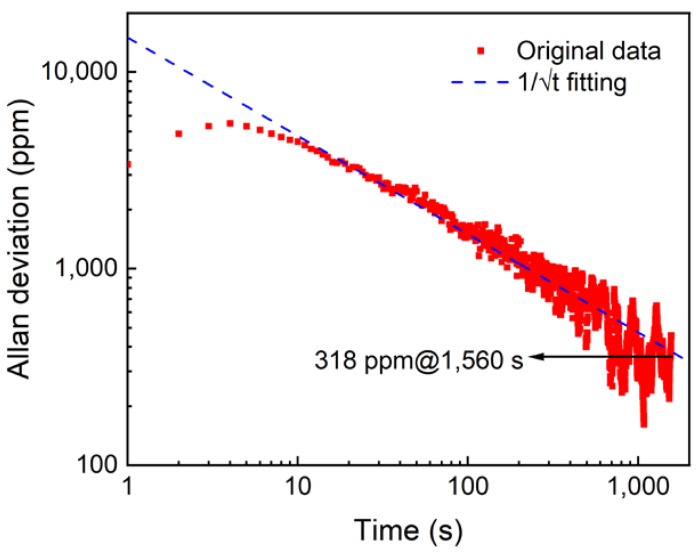
Allan variance analysis of the reflector-enhanced O_2_-PAS sensor system.

The Allan variance analysis method was used to investigate the system’s stability and detection limit. The collection points were obtained for continuous monitoring of 2.5 h when the photoacoustic cell was filled with pure N_2_. The processing results are shown in Figure 9. It can be seen that Allan deviation is closely related to 1/√t over a long period of time. This result proves that the noise in the measurement over several hours is mostly white noise. When the integration time is 1560 s, the detection limit can reach 318 ppm.

**Figure 10:**
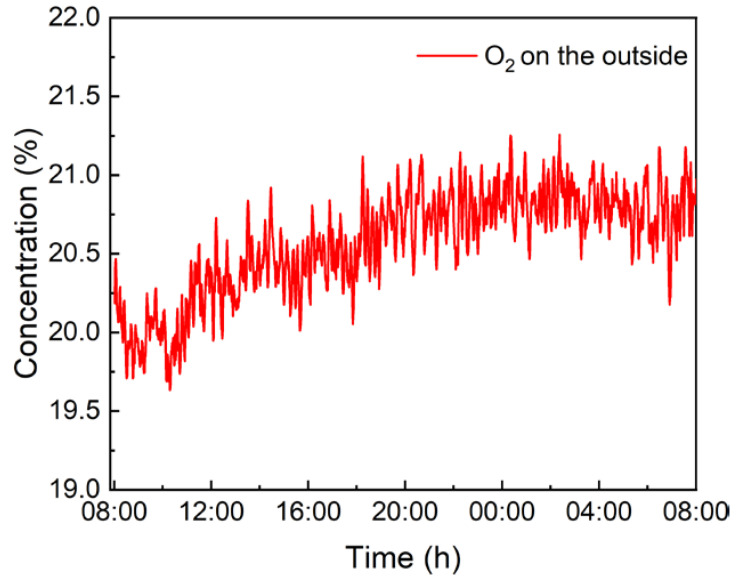
Real-time monitoring of O_2_ concentration on the outside for 24 h.

Finally, the O_2_ concentration on the outside was continuously monitored for 24 h. A gas pump was used to transport outdoor air to the photoacoustic cell. The obtained results are shown in Figure 10, which proves that this reflector-enhanced O_2_-PAS sensor system has excellent measurement ability for actual applications.

## 4. Conclusions

In this paper, a highly sensitive O_2_ sensor based on reflector-enhanced PAS is reported for the first time. Using a 760 nm diode laser as the excitation source, an O_2_ absorption line located at 13,144.54 cm^−1^ was selected. The diode laser beam was reflected by the adopted reflector. Therefore, a double-pass beam transmission was realized to increase the optical absorption. With such enhanced absorption, the O_2_-PAS sensor system had a signal improvement of 1.85 times when compared with the PAS system without the reflector. The reflector-enhanced O_2_-PAS sensor showed an excellent linear concentration response when O_2_ with different concentrations was used. The MDL was found to be 0.54%. The system stability was analyzed by using Allan variance, which indicated that the MDL for such a reflector-enhanced O_2_-PAS sensor could be improved to 318 ppm when the integration time of this sensor system is 1560 s. Finally, the O_2_ concentration on the outside was continuously monitored for 24 h, proving that this reflector-enhanced O_2_-PAS sensor system has excellent measurement ability for actual applications.

## Data Availability

The data presented in this study are available on request from the corresponding author.
